# Global partnerships in governing labour migration: the uneasy relationship between the ILO and IOM in the promotion of decent work for migrants

**DOI:** 10.1007/s43508-021-00022-x

**Published:** 2021-07-27

**Authors:** Nicola Piper, Laura Foley

**Affiliations:** School of Law, Queen Mary University of London, Mile End Road, London, E1 4NS UK

**Keywords:** Global migration governance, Decent work, Global partnerships, International organisations, IOM, ILO

## Abstract

This paper examines the multi-actor and multi-sited character of global labour migration governance as a sphere in which various organisations seek influence on the direction of global policy via various methods. We focus on the relational dynamics between the two key organisations which engage in the governance of labour migration, yet which have fundamentally different mandates and modes for governing: the ILO and the IOM. This paper contributes to the existing literature on global migration governance and the role of international organisations by applying the concept of ‘global partnerships’ to our examination of the relationship between those two key international organisations in the field of migration. We characterise the evolving ILO–IOM global partnership as an uneasy alliance along a “competition/clash-cooperation spectrum” and argue that, in order to manage the competing-cooperating dynamics, a type of strategic ILO–IOM partnership has emerged, an alliance which has also been driven by the blurring of public and private realms in new global migration governing forms and formats. The ultimate question raised by these developments is whether this global partnership will promote or obstruct the advancement of the decent work policy agenda for migrant workers.

## Introduction

Within policy circles, labour rights have come to be framed as “decent work” to specifically address the persistent problems and vulnerabilities of low-skilled, low-waged and informal sector workers, a considerable proportion of whom are women and migrants (Hauf, 2018; Standing, 2008).^1^ Promoting decent work has been part of the International Labour Organisation’s (ILO) mandate in various forms and formulations since its inception in 1919 as the first and thus oldest specialised agency of the United Nations (UN). However, it was not until 1999 when the ILO launched its “Decent Work” agenda that decent work became the organisation’s overarching frame and streamlined policy objective. As a result of this launch, the ILO reinstated its role as the key international organisation in charge of the world of work and employment in an era of accelerated neoliberal economic globalisation accompanied by rising inequality within and between countries. The manner of its reinstatement, however, did not occur without controversy, such as the Decent Work agenda’s implications for the advancement of labour standards (Hauf, 2018; Vosko, 2002).

The ILO is also a key player in the area of labour migration. Historically, it was the sole international organisation in charge of migration (Karatani, 2005), but since the end of World War II this policy field has gradually incorporated other global actors, depriving the ILO of its status as the sole international organisation concerned with the cross-border mobility of workers and the protection of their labour rights. Moreover, the global governance of migration no longer solely involves the UN and its related agencies, but now also encompasses extra-UN processes such as the Global Forum for Migration and Development (GFMD). This ‘broadening’ of the global migration governance space has opened up ample opportunity for participation by actors that were hitherto less or uninvolved, e.g. business groups—and the International Organisation for Migration (IOM), an intergovernmental organisation which had for long been operating at the margins of the UN system but not from within it. In recent years, the IOM has taken centre stage in the global migration governance arena through its role as coordinator of key global processes, promoting a ‘management’ approach to migration. These developments—multiplication of actor involvement in, and shifting sites of, global migration governance—as we argue, have hampered the promotion of decent work for migrant workers.

Although global migration governance, and the role of international organisations (hereafter “IOs”) within it, has become subject to flourishing academic studies, the relationship between the ILO and IOM has not been investigated in much detail. In our view, the analysis of their interaction cannot be grasped by a classic comparative analysis (as done by Fanning & Piper, 2021; Geiger, 2018; Geiger & Pécoud, 2020; Grugel & Piper, 2007; Kneebone, 2010; Pécoud, 2018) but calls for an alternative approach: a relational perspective. The focus on the relational dynamics between these two IOs allows us to go beyond an analysis of their roles as parallel functioning entities, as this is no longer sufficient since the IOM’s formal assimilation into the UN in 2016. Instead, we will show that a specific type of relationship between the ILO and the IOM is developing which we refer to as a ‘global partnership’. This strategic partnership, as we will show, has gradually evolved between these two organisations since the mid-2000s, due to political and institutional changes driven by, or surrounding, the UN. In substantive terms, as partnerships are often formed based on common areas of interest, this paper uses the example of each organisation’s initiative in the area of recruitment to shed light on the dynamics of their relationship as global partners, and we conclude by examining what this means for promoting decent work for migrant workers. The reason for focusing on recruitment is that “decent work is very clearly [an issue] that can be achieved *only* if there is fair recruitment” (ILO Representative, October 2020, emphasis added), and both the ILO and IOM have launched regional and international initiatives in relation to fair and ethical recruitment which have been fed into global governance processes and key outcomes such as the Sustainable Development Goals (SDGs) and 2018 Global Compact for Safe, Orderly and Regular Migration (GCM).

Methodologically, in its aim to unpack the relationship between the ILO and IOM from the viewpoint of global migration governance as a multi-actor and multi-sited sphere, this paper is based on an examination of policy papers, reports, press releases media coverage, and the international institutional framework relating to labour migration, covering the period 2000–2021. We also analysed an array of public statements, documents and resources issued by the ILO and IOM, and include observations made by one of the authors who participated in key global fora in-person^2^ and engaged in informal conversations with staff from the ILO and IOM. In addition, we analysed the content of a range of presentations given by ILO and IOM representatives on the subjects of recruitment and decent work which were available via 32 publicly accessible webinars held between April 2020 and April 2021.

The paper starts by embedding our argument within academic scholarship in relation to global partnerships in global governance. This is followed by an analysis of key shifts in the institutional and political environment that have led the ILO and IOM to take steps towards partnering, despite the very different nature of their respective mandates and organisational set-ups. This is followed by an examination of the ILO’s and IOM’s respective work on a specific policy area of decent work for migrants—recruitment—to illustrate the evolving competition/clash-cooperation dynamics of the ILO–IOM relationship. The paper concludes by exploring how the ILO–IOM strategic partnership could impact the promotion of decent work for migrant workers.

## The role of global partnerships in global governance

Our examination of the relationship between the IOM and ILO is conducted through the analytical prism of ‘global partnerships’. Scholars of global governance have conceptualised global partnerships largely as a *public–private* phenomenon^3^ or one that centres on “state-non-state partnerships” (Dingwerth & Hanrieder, 2010) which, in terms of public policy, are “cooperative initiatives that expand the political authority of non-state actors” (Bexell & Mörth, 2010: 6). These include both formal partnerships, such as those governed by a binding agreement between partners (i.e. in the form of Memoranda of Understanding (MoUs)), and informal partnerships. Such partnerships can fulfil an array of functions ranging from those centred on global standard-setting, rule-making and policy development, to collaborations which are more operational, those which are driven by advocacy, or those which seek to mobilise available resources in order to maximise outputs and impact (Bexell & Mörth, 2010). These partnerships are also often distinguished along certain types, such as those which ‘co-opt’ private actors, those which ‘delegate’ certain tasks to private actors, and those which ‘equally involve’ private actors as partners (Börzel & Risse, 2005; Korab-Karpowicz, 2020). Academic studies on global partnerships, and their effects, have examined public–private partnerships both in general (e.g. Andonova, 2017; Börzel & Risse 2005) and in relation to specific policy fields such as global health policy (e.g. Bexell, 2013) or environmental governance (e.g. Kramarz, 2013). What has been less looked at is the role of global partnerships between specific IOs and how they promote or obstruct the advancement of certain policy agendas, such as decent work for migrants.

Since the late 1990s, the need to strengthen global migration-related partnerships has been noted in global discussions, fora, and instruments relating to international migration in its relation to development.^4^ Academic studies examining migration partnerships have mainly focused on the public–private character of such partnerships (e.g. Bisong, 2015; Kunz et al., 2011), with some research highlighting the role of IOs, such as the IOM, in developing migration partnerships with states (Geiger, 2018; Kunz, 2013; Potaux, 2011). Scholars have also probed into the IOM’s partnership with local communities (Korneev, 2018), and the IOM’s relationship with other IOs, for example UNHCR (Elie, 2010; Koch, 2014). By contrast, the role of the ILO in migration partnerships has so far been overlooked, as has the nature of the ILO–IOM relationship as a form of global partnership.

Since we relate the ‘global’ in our discussion of migration governance to the relationship between IOs, we theorise the evolution of the ILO–IOM relationship by building on the specific reconceptualisation of the understanding of ‘public’ and ‘private’ partnerships proposed by Dingwerth and Hanrieder (2010). They advocate for the ‘public’ and ‘private’ elements in global politics to be redefined as concepts which are “based on two distinct modes and techniques of governance, or different normative spheres, as opposed to being primarily understood as the specific characteristics of actors” (Bexell & Mörth, 2010: 11). Dingwerth and Hanrieder (2010) note that, in public–private-partnerships, the prevailing understanding of ‘public’ and ‘private’ is based on the assumption of a neat dividing line between public actors acting ‘in the public interest’, while private actors are acting ‘in their own private interest’. In reality, however, the separation of ‘public’ and ‘private’ actors and spheres along these lines has become increasingly blurred (ibid). Dingwerth and Hanrieder (2010), therefore, suggest a new conceptualisation of ‘public’ and ‘private’ in which different organising principles separate the ‘public’ and ‘private’ sphere. They propose that the ‘public’ normative sphere should be understood as the realm in which collectively binding decisions are made, while the ‘private’ sphere should be understood as the realm in which both for-profit and non-profit individuals and organisations choose, within the confines of the respective legal regulations, their own terms for their interactions (ibid: 82). We shall relate this in the empirical section of this paper to the policy issue of “fair” or “ethical” recruitment in relation to which the ILO has contributed normative guidelines and the IOM is working with business actors.

Applied to our case, we argue that the ILO’s manner of operating can be associated with the “public normative sphere” in which binding decisions are collectively arrived at as the result of tripartite discussions and negotiations. Whereas the IOM’s decentralised, *non*-standard-setting nature which is open to any form of interaction, coupled with its highly projectised nature, puts it squarely into the category of an organisational actor which is open and prone to operating in the private sphere. According to a colleague, “when ILO speaks, it speaks also for business; when IOM speaks it tries to seduce business” (personal communication, February 2021) (see also Hennebry et al., 2018; Hennebry & Piper, 2021).

Yet, recent institutional changes within the arena of global migration governance have led to a blurring between public and private sphere, and in so doing, have provided a platform to both the IOM and ILO. These changes include a diversification of governing formats and a multiplication of actors due to the emergence of new global governing groups and processes^5^ (in which private interests are increasingly represented). We argue that these shifts have, on the one hand, spurred inter-institutional competition, but, on the other hand, these new governing spaces have promoted inter-institutional cooperation, and have provided arenas for increased interactions and dialogue between those two IOs. Importantly, these changes in global migration governance have set in motion a process which has pushed the ILO and IOM to step into, or at least engage with, the other migration organisation’s main realm. We argue that these developments have contributed to the genesis and evolution of a strategic global partnership between the ILO and IOM which we interpret as an ‘uneasy alliance’.

By introducing a relational perspective to the dynamics between IOs, our analysis moves beyond scholarly investigation of singular IOs in isolation or in comparison (e.g. Betts, 2011), or in the context of specific global processes such as the GFMD and the GCM (e.g. Ferris & Donato, 2019; Hennebry & Piper, 2021). In contrast, our examination of the development of a global partnership between the ILO and IOM places the emphasis on how they relate to one another and how they navigate and assert their mandates within the evolving global migration governance arena. We argue that the ILO–IOM dynamics are played out along the “competition/clash-cooperation” spectrum wherein both competition and cooperation exist simultaneously. In order to manage the competing–cooperating dynamics between them, we contend that the ILO and IOM have developed a global partnership at the intersection of the public normative sphere (where collectively binding decisions are made), and the private sphere (where individual organisations choose their own terms of interaction). The next section discusses how despite the contrasting mandates and institutional designs of the ILO and IOM, a combination of shifts in global migration governance since the early 2000s has altered the relationship between the ILO and IOM.

## Instituting migration as a global governing sphere

The ILO and IOM are undoubtedly the two key institutional actors at the global level in relation to international labour migration today, but this has not always been the case. The IOM was not originally established with a mandate to deal with labour migration^6^ but instead its historical purpose was to provide a range of migration management services to states which include a wide variety of programmes relating to border management and assisting people displaced by crises (Geiger & Pécoud, 2010; Hall, 2015). Over time, the IOM has expanded the range of migration-related policy angles so much so that its portfolio has come to essentially cover all aspects of migration, including migration in relation to development, climate change, and labour migration. This is evident from IOM’s wide array of programmes and expanding institutional divisions from the late 1990s onwards which has resulted in its omnipresence.^7^

In contrast, the ILO’s key role has been to develop and promote labour standards^8^ and has thus been a norm-setting^9^ organisation ever since its inception in 1919. Importantly, the ILO’s standard-setting and central decision-making process involves tripartite deliberations which include not only governments, but also employers’ and workers’ organisations, resulting in a high level of consensus and legitimacy. The emphasis in the design of the ILO’s policies and programmes is on promoting decent work for all workers, including migrants, via sector- or group-specific instruments, and also in relation to explicit problems such as violence at the workplace or global supply chain dynamics.

The IOM, by contrast, was born out of the post-Second World War refugee situation as a US-dominated organisation whose role was to assist governments in managing the cross-border movement of people. With the collapse of the Soviet Union and the fall of the Berlin War, the IOM had to gradually reinvent its role. As an almost entirely projectised intergovernmental organisation that is run by and for governments, *any* fundable aspect of migration became of interest. This project-based funding model^10^ is subject to ongoing criticisms amidst concerns that influential states use their ‘donor power’ to engage the IOM to perform dubious ‘migration management’ services^11^ (see also Crépeau & Atak, 2016; Pécoud, 2018). While the IOM’s decentralised structure has assisted the organisation in building up an all-round portfolio of projects by providing the necessary agility and flexibility to act fast upon donor demand, its projectised nature has also led to short-termism and lack of an overarching strategy. The ILO, by contrast, has a solid core budget which allows it to focus on strategic planning and long-term programming. It has the benefit of not having to assert its expertise via a massive publicity machinery to the same extent that the IOM has had to secure its temporary project-based funding.

The historical evolution of the multi-actor nature of global migration governance, as we argue, has caused the relationship between the ILO and IOM to initially be characterised mainly by competition, as both organisations sought to assert themselves as the lead on labour migration issues. This competition is rooted in the fact that the creation of the IOM shattered the ILO’s ambition to remain the sole IO in the labour migration sphere and curtailed the expansion of the ILO’s portfolio on international migration (Fanning & Piper, 2021). The competitive nature of their relationship is particularly evident on the global political arena^12^ where principles and foci of policy are decided and therefore the stakes are high (participant observation by first author). However, as we argue next, key shifts in the institutional and political environment over the past two decades have altered the nature of the ILO–IOM relationship and have produced space for the formation of an inter-institutional partnership in relation to an array of specific policy areas of labour migration.

### ‘Migration’ partnerships within and beyond the UN

Since the late 1990s, the UN has pushed for alliances and partnerships to develop among its specialised agencies, and between UN entities and external organisations (see UNGA, 1997, 2002).^13^ With the turn of the millennium, as migration gained increasing prominence on the global agenda,^14^ a multiplication of actors, groups and fora populated the ‘global migration governance arena’. As a result, IOs were provided with new opportunities to interact on migration-related issues, and inter-institutional partnerships were fostered. For instance, the Global Commission on International Migration (GCIM) was created in 2003 to take the global migration agenda forward through conducting an analysis of gaps in approaches to migration.^15^ One of its key recommendations was that a high-level inter-institutional group should be formed within the UN comprised of agencies involved in migration-related activities.^16^ The Global Migration Group (GMG) was subsequently established in 2006 by the heads of the various UN agencies (including the ILO), in addition to the IOM. The GMG was built upon an existing inter-agency group (the Geneva Migration Group^17^) mandated to coordinate international migration efforts.

The following year, 2007, another important milestone was reached in the global migration governance arena: the launch of the GFMD. The establishment of this state-led forum was engineered by the UN Special Representative for International Migration, Peter Sutherland, and his team in recognition that states can only be enticed to sit around the table to discuss migration in an informal, non-binding, non-UN setting (personal communication with former UN staff, April 2021). Thus, as a state-led platform, the GFMD provides governments a space to discuss and build trust on a sensitive policy area characterised by its political sensitivity. Although this forum takes place outside of the UN, it is connected to the UN system through the role of the UN Special Representative for International Migration (ILO, 2014). The need to strengthen global partnerships has been ingrained in the GFMD’s discussions since the forum’s inception^18^ and, in 2010, the forum launched a specific “Platform for Partnerships” mechanism to foster the development of partnerships.^19^ Another key milestone, the 2018 Global Compact on Migration (GCM),^20^ has instituted migration-related partnerships both through its Objective 23: “Strengthen international cooperation and global partnerships for safe, orderly and regular migration”, and via its establishment of the “UN Network on Migration” (comprised of a group of UN agencies) to support the implementation of the GCM.

The aforementioned ‘sites’ of migration governance represent new governing forms and formats in which a blurring of the public and private sphere has occurred. While the UN is a normative organisation whose conventions are legally binding and whose core members are states, in the new migration governance arenas that it has developed or promoted, such as the GFMD and GCM, private interests have increasingly been represented via the expanding participation of private sector and business groups, narrowing the space for (human/labour) rights advocates^21^ (Hennebry et al., 2018; Hennebry & Piper, 2021). The GFMD even established a Business Mechanism in 2015 to form part of its annual forum. The IOM has positioned itself perfectly to move into a coordinating role of such key global processes which is part of its move into the UN system.

### IOM as “UN Migration”

A second key shift that has contributed to the development of a strategic ILO–IOM partnership has been the IOM’s formal incorporation into the UN. While the IOM was historically situated outside of the official UN system, it has always worked closely with, and been adjacent to, the UN since it was established in 1951 (UN News, 2016).^22^ Since the early 2000s, a number of significant steps have been taken to deepen and formalise the UN–IOM relationship, which we argue has, in turn, shaped ILO–IOM relations. Following the establishment of the GMG in 2006, the IOM increasingly cooperated with other UN agencies to implement programmes relating to migration.^23^ This has led to specific inter-institutional collaborations, for example, the development of ILO–IOM labour migration-related projects at the regional and national levels.^24^

Lebon-McGregor’s (2020) research sheds light on the factors that motivated the IOM to become formally integrated into the UN. An IOM representative whom Lebon-McGregor interviewed stated that when the 2002 Doyle Report proposed that an agency *within* the UN could be made responsible for migration, this led the IOM to “smell competition” and, thus, incentivised the organisation to strategically “get closer to the UN” (2020: 168) in order for it to stay important on the global stage. In 2016, the UN-IOM agreement was signed which officially incorporated the IOM into the UN system as a “related agency”.^25^ The IOM Director General at the time, stated that “becoming a part of the UN family will give IOM a vital voice at the UN table” (UN News, 2016). Rother (2020: 9) noted that the other UN entities (of whom there are 38 that work in the migration field^26^), protested against the IOM being officially heralded by the UN as “the global leading agency on migration”.^27^ However, the IOM has since rebranded their official communications to include the term “UN Migration” underneath the organisation’s logo.

Migration and legal scholars have comprehensively outlined what led up to the signing of the UN–IOM agreement, what its impacts are, and why the IOM opted for ‘related’ as opposed to ‘specialised’ agency status (e.g. Geiger, 2020; Grant et al., 2017; Lebon-McGregor, 2020; Rother, 2020).^28^ For our purposes, the most interesting aspect of the updated relationship is that the IOM is still able to retain its independence which, as highlighted by Geiger (2020), was something that the IOM’s Member States were keen to preserve and thus pushed for the IOM to only become a UN ‘related’ agency. This distinction means that the IOM has no official reporting obligation to the UN and that it can operate with greater freedom and flexibility than other UN migration-related agencies (Grant et al., 2017; Hennebry & Piper, 2021).^29^ There are also ongoing criticisms that IOM’s status as a non-normative^30^ organisation without a human rights or labour rights protection mandate calls into question its commitments towards migrants’ rights (Crépeau & Atak, 2016; Pécoud, 2018).^31^ However, one of our informants (personal communication, March 2021) stated that the IOM’s UN affiliation means that it had already become informally bound to adhere to the UN’s norms and that its formal incorporation into the UN has helped to pave the way for IOM to foster partnerships with other UN entities.^32^

The IOM’s formal assimilation into the UN has been accompanied with it taking on an even more central role in global governing processes. For example, the IOM was tasked with “servicing the negotiations” for the GCM (UNGA, 2016: 23). This builds on the role that the IOM has played since 2006 as the GFMD’s closest institutional partner and host of the GFMD Support Unit.^33^ The IOM’s leading role in global migration governance was further cemented when the GCM stipulated that the IOM would act as Coordinator and Secretariat of the new “UN Network on Migration”. We argue that the IOM was chosen as coordinator of the aforementioned global governing mechanisms for a range of reasons relating to the nature of the IOM and the evolution of these governing processes. Since the mid-2000s, migration governing formats and sites have multiplied and evolved to also incorporate private actors, and the UN has blurred into a public *and* private sphere which centres not only on public good creation but also on engagement with the private sector. Thus, we argue that the IOM was selected to take on a convening and coordinating role in these blurred governing spaces as it is a flexible, non-normative, decentralised organisation with a predisposition to operating in the private sphere and liaising with business groups.

In contrast, the ILO has faced challenges in reasserting its leadership in global migration governing processes. From the late 1990s, as the ILO moved towards becoming increasingly reliant on producing “soft” instruments with relatively lower degrees of precision and obligation (Jakovleski et al., 2019),^34^ this also impacted its labour migration-related outputs. For example, when the ILO Governing Body mandated the organisation in 2004 to develop a rights-based approach for managing labour migration, instead of developing new legally-binding obligations or promoting the ratification of its two existing migrant worker conventions,^35^ the ILO instead launched a *non-binding* Multilateral Framework on Labour Migration in 2006 in which states’ “sovereign right… to determine their own migration policies” was explicitly recognised (ILO, 2006: vi).^36^

Notably, at the 2011 GFMD, the ITUC General Secretary criticised the existing shortfalls in the global migration governance system and explicitly called for the ILO to occupy a more central position in governing processes, stating that the ILO “was not doing enough” (ICMC, 2011: 25). In 2014, following the launch of the ILO’s “Fair Migration Agenda”^37^ and its Fair Recruitment Initiative—with which labour migration was placed at the forefront of the Decent Work Agenda^38^—the ILO appeared to indicate that it was “now willing to take a leadership role on the rights of migrant workers” (Crepéau & Atak, 2016: 131). The ILO was able to achieve the inclusion of a separate goal on decent work (Goal 8) in the SDGs and was able to contribute to the GCM negotiation processes.^39^ It also is an Executive Committee Member of the UN Network on Migration and a co-lead (alongside IOM) of the network’s working group on Bilateral Labour Migration Agreements. Yet, the ILO is paid lip service as joint partner in the Working Groups’ coordination and also not given equal footing compared to the IOM in relation to the Network: our reading of the inward operational document and outward ‘facing’ website^40^ is that the IOM is designated as coordinator of the network, while the ILO is only mentioned in relation to its instruments, hence not as an actor with a specific role in the network. Thus, the ILO is faced with considerable barriers when trying to reassert its leadership role in relation to the cross-border mobility of workers, especially in the case of irregular or undocumented migrants since it takes a labour protection approach applied to *all* workers, unlike the criminalising tendency of the “migration management” approach associated with the IOM. The barriers experienced by the ILO are also partially due to the ILO’s institutional design and mandate which prevent it from performing a coordinating role in ‘blurred’ global governing spaces (such as the GFMD) in which private actors have been increasingly incorporated and influenced the agenda. To legitimately take up a coordinating role in these governing spaces, the ILO would have to obtain authority through its tripartite decision-making procedure.

The IOM’s leadership role in the global migration governance arena (and the ILO’s apparent side-lining) has wider ramifications beyond the sense of competition that it fosters between the ILO and IOM as it has consequences for the promotion of decent work for migrant workers. For example, the ILO’s much smaller presence at the GFMD, compared to the IOM, has meant that the topic of ‘decent work’ in relation to the drivers of migration has received a lack of attention (Bingham, 2019). However, in relation to the cooperation carried out under the ILO–IOM global partnership on fair recruitment, as we will show below, the ILO is moving out of the shadows.

### The ILO–IOM memorandum of understanding

To manage the complex competitive side of the ILO–IOM relationship, as we argue, a formal agreement was needed to define their collective, and respective roles, in the governance of labour migration, and to determine the conditions under which they could or should collaborate. In October 2020, a key milestone in ILO–IOM relations was reached when the two organisations signed a partnership agreement in the form of a Memorandum of Understanding (MoU). The plan for an agreement to be signed by the two IOs had been in the pipeline for a number of years^41^ and its stated purpose was to provide a framework for broad cooperation on labour migration issues.

The text of the MoU underlines that the two IOs have a pre-existing partnership but that, by signing the formal agreement, the aim is to strengthen this partnership (and thus deepen international cooperation) by building “on complementarities, comparative advantages and added value whilst also avoiding duplication” (IOM, 2020a, 2020b, 2020c, 2020d: 3).^42^ While the text of the agreement underlines that it aims to *strengthen* the already existing partnership between the two IOs, we interpret the ILO–IOM MoU as more of a tool to help strategically *manage* their complex relationship. At the time of writing this paper, the work plan of the ILO–IOM agreement had not yet been finalised (and thus is it is difficult to know what specific outcomes will arise from it), but we envision that the MoU will be used as a mechanism for cooperation on identified key migration policy areas, such as recruitment.

We conceptualise the ILO–IOM global partnership as one which blurs the public and private spheres. This is because, on the one hand, we associate the ILO’s manner of operating with the “public normative sphere” due to its standard-setting mandate and its decision-making procedure based on collective tripartite discussions with the involvement of trade unions. While, on the other hand, we associate the IOM with the private sphere due to its highly projectised nature, ad hoc decision-making, and itspredisposition to liaising with private actors. In addition, both IOs participate in ‘blurred’ governing spaces such as the GFMD and the UNMN. This global partnership benefits both organisations as it lends a level of legitimacy to the IOM while it better connects the ILO (and through it, trade unions) to the new arenas of migration governance which increasingly represent private interests. At the same time, it is born out of a political and institutional context of competition between two very different organisations which therefore renders this partnership an uneasy alliance.

The next section illustrates these points by our examination of both organisations’ fair recruitment initiatives which demonstrates the ways in which their strategic partnership manifests.

## Decent work for migrants through global partnerships: fair recruitment

The example of the ILO’s and IOM’s initiatives aimed at improving the fair and ethical recruitment of migrant workers lends itself nicely to show how the ILO–IOM dynamics are played out in the form of an uneasy alliance along the “competition/clash-cooperation” spectrum. Fair recruitment^43^ processes are an issue that has received increased attention as migration gained prominence on the global agenda. Recruitment has formed part of the GFMD’s itinerary since the first summit in 2007 which included sessions on protecting migrants from abusive recruitment practices. Some of the first summit’s outcomes included how to foster standardised systems and codes of conduct for recruitment and how to regulate recruiters and other actors involved in hiring and placing workers overseas. These issues have continued to gain traction at the GFMD and other fora in the intervening years,^44^ and the 2013 High-level Dialogue on Migration and Development saw participating states agree to develop global standards for migrant workers’ recruitment practices (Newland, 2013). We argue that the recruitment-related initiatives developed by the ILO and IOM have often stood in competition with each other, but, at the same time, promoting fair recruitment has become one of the key areas upon which the ILO and the IOM have been cooperating and it is poised to constitute part of the range of activities addressed by their newly formalised partnership.

### Competitive phase: launching of individual initiatives

Within the past decade as the IOM moved closer to the UN, it began to implement an increasing range of initiatives and partnerships aimed at promoting the *ethical* recruitment of migrant workers, the most notable of which is IOM’s *International Recruitment Integrity System* (IRIS) launched in 2014 with the International Organisation of Employers (IOE). This initiative, known as “IRIS: Ethical Recruitment”, is the IOM’s flagship international programme to promote the ethical recruitment of migrants and it involved creating recruitment principles—the “IRIS Standard”—that define what ethical recruitment means in practice and how labour recruiters can show compliance. The ILO’s international labour standards formed an integral part of the discussions surrounding the development of IRIS and the ILO’s standards, alongside international human rights instruments, formed the foundation of the global principles which IOM created. Through its IRIS initiative, the IOM also launched a voluntary accreditation mechanism in 2018 through which private international recruitment agencies can apply to be recognised as fair recruiters.^45^

In the same year as IOM launched IRIS, the ILO launched a global “Fair Recruitment Initiative” (ILO-FAIR) which they state was driven by: (1) the increasing volume of requests that it had received from its tripartite constituents to provide further guidance on what constitutes fair recruitment practices, and (2) recognition that a key part of its Decent Work Agenda is to promote fair recruitment (ILO, 2014: 23). The aims of the ILO initiative include protecting workers’ rights, reducing the cost of labour migration, and protecting workers from exploitative recruitment practices (for more detail, see ILO, 2017b). This initiative is premised on social dialogue and is implemented in close collaboration with multiple stakeholders including the ILO’s tripartite constituents (governments, workers’ organisations, and employers’ organisations).^46^ Under this initiative, the ILO published its *General Principles and Operational Guidelines on Fair Recruitment* in 2016.^47^ The13 general principles outlined in the non-binding guidelines were developed in consultation with the ILO’s tripartite constituents and were derived mainly from international labour standards and related ILO instruments (ILO, 2019a).^48^ The ILO’s Fair Recruitment Initiative also supported the 2018 launch of the Migrant Worker “Recruitment Adviser” Platform^49^ by the ITUC, a mechanism which stands in competition with IOM’s IRIS initiative.

In 2019, the IOM also set out to develop and publish its own guidance relating to recruitment. The content of IOM’s guidance was generated through discussions held at the first-ever *Global Conference on the Regulation of International Recruitment and Protection of Migrant Workers* in Montreal, Canada, which IOM co-hosted.^50^ At this conference, the dialogue centred upon “co-creating clear, practical guidance to better monitor the private recruitment industry and protect migrant workers throughout recruitment, deployment and employment” (IOM, 2019). The subsequent conference output—“The Montreal Recommendations on Recruitment: A Roadmap towards Better Regulation”—was published by IOM in 2020 and consisted of 55 recommendations that had been articulated by those who participated in the conference (IOM, 2019, 2020a).^51^

### Clash: contrasting standard-setting mechanisms

We conceptualise the IOM’s move into standard-setting as going beyond ‘competing’ with the ILO thereby amounting to ‘clashing’. This ‘clash’ stems from the ILO and IOM’s notably different mandates, institutional designs, and approaches to regulation. State regulation is the realm of the ILO but the IOM has moved into ‘private’ regulatory space by developing voluntary codes of conduct and voluntary certification schemes. While IOM’s IRIS Standard, its definition of ethical recruitment, and its Montreal Recommendations are based on ILO standards (partly for credibility reasons), the ways in which IOM’s standards and guidance were developed only appear similar to ILO’s standard-setting processes (i.e. multiple stakeholder involvement), yet there is a stark difference in relation to *who* is consulted.

IOM’s IRIS Standard was developed through consultations “with state and business actors”^52^ and IOM’s Montreal Recommendations arose from two days of discussions between policymakers, experts, and practitioners from 30 states, alongside IOs.^53^ In contrast, the ILO’s standards and guidance are developed through tripartite negotiations between its constituents—governments, workers, and employers—who provide input not only into the discussions but also to the written output.^54^ Even if desired by the IOM, it may take some persuasion to get the ITUC and global union federations to participate in an IOM-led initiative of this kind.^55^ Furthermore, it warrants noting that the IOM’s move into the realm of standard-setting in relation to recruitment raises concerns with regards to legitimacy.^56^

### Cooperation: partnering on recruitment-related events

The preceding sections have analysed how the ILO’s and IOM’s individual initiatives, and the written guidance associated with them, have developed seemingly in competition/clash with one another. However, since the 2014 launch of each initiative, the ILO and IOM have *also* cooperated and partnered on recruitment-related events. Hence, we argue that the ILO–IOM dynamics are characterised by the *simultaneous* existence of both competition/clashes and cooperation. For example, at the 2019 Montreal conference which IOM co-hosted, and from which IOM published its Montreal Recommendations, the IOM gave the ILO a prominent role, and the ILO’s recruitment-related standards and guidelines were cited throughout many of the conference’s discussions (IOM, 2019).

The ILO and IOM have also co-organised workshops on the fair and ethical recruitment of migrant workers, for instance in Guatemala in 2019 in which representatives from government institutions, the ILO, and the IOM were brought together with employers, workers, and CSOs (ILO, 2019b). The ILO and IOM have also partnered at the Global Forum for Responsible Recruitment. This forum was launched in 2017 by the Institute for Human Rights and Business (IHRB), supported by the IOM, but, over time, it has increasingly involved the ILO with 2021 marking the first time that the ILO co-hosted alongside the IOM and IHRB. This forum is a new format which blurs the public and private spheres. It primarily originated as a business-focused forum—bringing together global brands, recruitment agencies, and suppliers, alongside IOs and CSOs—however, ILO’s involvement in 2021 meant that not only were labour unions able to have a greater presence at the forum, but so too was the issue of decent work (IHRB, 2021). The ILO and the IOM have used such forums and workshops to both promote their own recruitment initiatives and to promote their “strategic alliance” (ILO, 2019b; IHRB, 2021).^57^

The IOM in particular has endeavoured to promote both the complementary and collaborative nature of the ILO’s and IOM’s respective recruitment initiatives. For example, the IOM has stated that “within the UN system, IOM and ILO are working together to promote ethical and fair recruitment” (IOM, 2020c: 2). However, at the same time, the IOM has sought to distinguish between each organisation’s individual recruitment-related roles, highlighting that while the ILO works on *policy* aspects through its Fair Recruitment Initiative and its General Principles and Operational Guidelines, the IOM focuses on *operational* aspects of ethical recruitment through its IRIS Initiative (ibid).^58^ The IOM has also endeavoured to underline how their 2019 Montreal Recommendations “build upon” and “complement” the ILO’s existing 2016 fair recruitment principles.^59^ The IOM have also emphasised, in light of the impact of COVID-19 on migrant workers, that they are committed to continuing working “very closely” with the ILO on recruitment-related issues.^60^

## The impact of the ILO–IOM partnership on decent work for migrant workers—concluding remarks

This paper has examined the relational dynamics between the ILO and the IOM, the two key organisations engaged in the global governance of labour migration yet who have fundamentally different mandates and modes of governing. By introducing a relational perspective to our examination of the dynamics between IOs in the context of migration policy, this paper contributes to the existing literature on global migration governance in relation to the development and expansion of global partnerships between IOs.

We have shown that the historic relationship between the ILO and the IOM is rooted in competition due to the different lenses through which these two IOs have traditionally approached international migration and its governance. However, we argue that from the early 2000s onwards, key shifts in the institutional and political environment at the global level have altered ILO–IOM relations. These shifts include: (1) the multiplication of actors, governing sites and formats in the migration governance arena; (2) increased inter-institutional interactions at various governing sites, and; (3) the IOM’s assimilation into the UN. These events have set the foundation for the UN’s “labour agency” (ILO) and “migration agency” (IOM) to develop a more concrete strategic partnership on the governance of labour migration, as the result, as we argue, of a blurring of the ‘public’ and ‘private’ spheres.

Having characterised the nature of the ILO–IOM relationship as an uneasy alliance along a competition–cooperation spectrum in which competition and cooperation simultaneously co-exist,^61^ this led us to argue that in order to better manage these dynamics, a type of strategic global partnership has evolved between the ILO and IOM. We used their respective fair and ethical recruitment initiatives to illustrate this by showing that, despite these programmes initially being conceived as ‘in competition’ with one another, over time, the ILO and IOM have strategically partnered on this policy area in ways which benefit both organisations.

We do not envision that competition between the ILO and IOM will disappear entirely given their ongoing mutual interests in, yet different approaches to, the governance of labour migration. However, we think that the competitive elements and the overlaps between these IOs will be better managed through the formal partnership agreement that they signed in 2020. We predict that the ILO–IOM partnership as outlined in the MoU will serve two functions. First, in our view it will be used as a tool to manage, if not prevent, *bad* competition, i.e. one that would otherwise result in duplication and possible contradictions in both organisations’ messaging to governments and other common stakeholders. Second, it will attempt to nurture *good* competition that draws on the IOM’s agility and entrepreneurial spirit on the one hand, and the ILO’s collective normative standards that are carefully negotiated via tripartite consultations and mechanisms on the other hand.

Yet one key question remains, what does the evolving ILO–IOM partnership mean for the promotion of decent work for migrants? ILO–IOM partnering could help the plight of migrant workers as increased collaborations with the ILO could shape how the IOM operates and could help place decent work for migrants on to the global agenda. Furthermore, the rising prominence of the IOM in the global migration governance sphere has pushed the IOM to reach out and engage with more non-state actors, such as CSOs.^62^ This, in turn, could benefit migrant workers as the IOM now interacts with an increased number of actors who represent the concerns of migrant workers. These interactions have also been amplified by ILO–IOM partnering on recruitment-related issues as direct engagement between workers’ organisations and the IOM has been fostered.^63^

Nevertheless, despite strategic ILO–IOM partnering, the increased centrality of the IOM within the multi-actor governing processes could also lead to questionable outcomes for advancing the decent work agenda for migrants. This is due to ongoing concerns surrounding the IOM’s ability to operate with greater flexibility than other UN migration-related agencies (e.g. Grant et al., 2017). Furthermore, as noted previously, the IOM’s move into the realm of standard-setting raises concerns with regards to legitimacy. These concerns relate not only to the ad hoc consultation processes through which IOM’s standards and guidance are formulated, but also to the question of whether the IOM has any legitimate channels to get involved in standard-setting given that it is a non-normative organisation without a human rights or labour rights’ protection mandate. Taking on the role of coordinator in global governing processes does not mean that the IOM will have changed much internally as it remains state-oriented and its institutional design and organisational mandate (in which promoting migrants’ rights is not enshrined) remains the same. Therefore, there are no guarantees that the standards which the IOM may generate will be geared towards advancing migrant workers’ labour rights with felt effects ‘on the ground’. At the end of the day, how the ILO–IOM partnership will pan out, and how this impacts upon the promotion of decent work for migrants requires more analysis. It will also likely depend upon how exactly the MoU will be implemented at the global, regional, and country levels.^64^ This space will have to be watched closely since the IOM–ILO global partnership is “work in progress”.

## Footnotes

1 This is also reflected in the United Nations (UN) Millennium Development Goals and their successors, the Sustainable Development Goals Agenda 2030 (as per their Goal 8).

2 Those are: the ILO conferences in 2004, 2010 and 2011; 9 of the 12 Global Fora on Migration and Development held up to 2020; the 2nd UN High Level Dialogue on Migration and Development in 2013, and various Global Compact on Migration consultations and adoption event.

3 Public–private partnerships were endorsed in UN General Assembly Res. A/56/76 (24 January 2002).

4 Most notably, the concept of partnerships was explicitly included in the Millennium Development Goals (MDGs) by way of Goal 8: “Develop a global partnership for development”, and its successor the Sustainable Development Goals via Goal 17: “Strengthen the means of implementation and revitalise the global partnership for sustainable development”.

5 For example, the GCIM, the GMG, the GFMD, the GCM, and the UN Migration Network.

6 The IOM’s constitution makes no reference to labour migration nor migrant workers.

7 IOM’s projects quadrupled between 1995 and 2014 (Thouez, 2013).

8 The ILO has created 190, and oversees more than 150, international labour conventions, in addition to protocols and recommendations.

9 Its normative activity is typically preceded by research and often followed up by technical assistance (Standing, 2008).

10 97 per cent of IOM’s resources rely on voluntary contributions, the majority of which are reserved for specific projects (MOPAN, 2019: 4).

11 Such as the IOM’s assisted “voluntary” return programmes.

12 This also relates to the differences between headquarter level relations (shaped by adherence to principles or macro-institutional mandates) and in-country relations (influenced by greater pragmatism related to the concrete implementation of programs and projects).

13 Kofi Annan’s 1997 and 2002 Reform Agendas sought to improve coordination within the UN system and to counter the overlapping and incoherence between UN entities.

14 The catalyst of this can be traced back to 2002 when UN Secretary-General Kofi Annan placed international migration on the global agenda as a key challenge which would need to be addressed in a more comprehensive, coordinated, and goal-oriented manner (see UNGA, 2002).

15 Their full mandate was to: (1) “place international migration on the global agenda”, (2) “analyse gaps in current approaches to migration and examine connections with other issue-areas”, and (3) “to present recommendations to the UN Secretary-General and to other stakeholders” (GCIM, 2003: 2).The Commission ran between 2003 and 2005 and it was the first global panel to address migration.

16 The GCIM recommended that a global migration group be created. See GMG (2021).

17 The Geneva Migration Group (established in 2003) had more limited membership, including the IOM, ILO, OHCHR, UN Conference on Trade and Development (UNCTAD), UNHCR and UNODC. The *Global* Migration Group expanded the membership to include UNDESA, UNDP, UNFPA, World Bank, UNESCO, UNICEF, UNITAR, UN Women, FAO, UNU and the UN Regional Commissions. See: https://www.iom.int/global-migration-group.

18 For example, at the first GFMD in 2007, one of the roundtable themes was on “Enhancing policy and institutional coherence and promoting partnerships”.

19 The GFMD’s “Platform for Partnerships” mechanism is a dual process that combines an online platform with face-to-face interactions to “encourage governments to work in partnership—with each other and/or with other key non-government stakeholders in developing and implementing migration and development policies and programs” (see https://www.gfmd.org/pfp).

20 This Global Compact was prepared under the auspices of the UN.

21 This development is not confined to the policy area of migration (see Kamat, 2004), as also evident from the UN Global Compact (Ruggie, 2003).

22 The IOM was granted Permanent Observer status to the UN General Assembly in 1992, and signed a cooperation agreement with the UN in 1996.

23 For example, between 2008 and 2012, the ILO and IOM have collaborated on the Joint Migration and Development Initiative, alongside the UNDP, UNHCR, UNFPA, and UN Women.

24 Especially at the country-level as, following the UN’s “Delivering as One” agenda, the ILO and IOM have undertaken joint field programmes. They have also developed collaborative projects in a range of regions including in Africa, the Arab States, Asia–Pacific, Europe, Latin America and the Caribbean. They have worked together on the Programme on Labour Migration for Development and Integration (JLMP), have co-authored an array of publications pertaining to labour migration, and have co-convened a Task Force on Migration and Decent Work that was launched by the GMG in 2013.

25 This took place at the UN General Assembly High-Level Summit meeting in 2016. The 2017–2018 MOPAN Assessment of the IOM notes that “the organisation’s accession to the UN system in 2016 offers new opportunities for strategic and operational engagement at country, regional and international levels” (2019: 3).

26 This is evidenced by the composition of the UN Network on Migration.

27 As such, the final text of the UN-IOM agreement stated that the UN recognises the IOM as “an organisation with *a* global leading role in the field of migration” (art. 2, para 1, emphasis added).

28 For example, IOM Member States were repeatedly unconvinced that the positives associated with of specialised agency status outweighed the negatives (e.g. IOM, 2003: 1), with fears that obtaining specialised status would mean giving up its independence.

29 The text of the UN-IOM agreement states that the IOM “shall function as an independent, autonomous and non-normative international organisation in the working relationship with the UN” (art 2, para 3).

30 For example, Sharan Burrow, the General Secretary of the International Trade Union Congress (ITUC) stated at the 2011 GFMD that “the IOM has no normative basis for work and no competency to oversee the implementation of states’ obligations under international migration laws” (ICMC, 2011: 25).

31 Although the IOM states that it “strives to protect migrant workers” (IOM, 2021).

32 Since moving closer to the UN, the IOM has also conducted a mapping exercise of civil society organisations (CSOs) with the aim of reaching out to relevant civil society actors to seek greater legitimacy. A concrete example is the MoU that IOM signed on 1st October 2020 with the Migrant Forum in Asia, a regional network of NGOs, associations, trade unions, lawyers, migrant workers and migrant advocates (IOM, 2020d).

33 GFMD (2021).

34 Since 1998, the ILO has since produced an increasing number of “soft” instruments (i.e. Recommendations, Declarations, and policy frameworks) all of which have relatively lower degrees of precision and obligation compared to legally-binding commitments (Jakovleski et al., 2019: para 9) and such mechanisms are a way to “to make labour protection more palatable” (ibid: para. 49) to governments.

35 The Migration for Employment Convention (Revised) no. 97 (1949) and the Migrant Workers (Supplementary Provisions) Convention no. 143 (1975).

36 Importantly, Tungohan (2015) also points out that the 2006 Framework lists the “effective management of labour migration” ahead of “migrant protection” in its hierarchy of priority areas.

37 See ILO (2014).

38 See ILO (2017b).

39 During the GCM negotiation processes, the ILO informed the UN Secretary General that it is “ready to play its role and to lead coordination of UN support for commitments adopted on decent work and labour migration, in cooperation with and complementing the work of other organisations like IOM” (ILO 2017a: 2, emphasis added).

40 For the inward looking document, see https://migrationnetwork.un.org/sites/default/files/docs/migration_mptf_ops_manual_rev_dec_2020_final.pdf; and for the outward ‘face’, see e.g. the website/Knowledge Hub (https://migrationnetwork.un.org/).

41 As per a range of UN informants that we spoke to for this paper.

42 The MoU states that it will help to enhance each organisation’s effectiveness and impact in pursuing their mandates and that this includes supporting their respective constituents in implementing the GCM. It also states that, by signing the agreement, the IOM and the ILO aim to “strengthen international cooperation, including support for the implementation of the SDGs, the GCM, and the ratification and implementation of relevant UN and ILO Conventions, Recommendations and Protocols, as well as other international standards related to migration governance and the rights of migrants” (IOM, 2020a, b, c, d: 4).

43 The term “fair recruitment”—which Wickramasekara and Baruah (2017: 24) note gained traction after its inclusion as one of the key themes in the ILO’s (2014) Fair Migration Agenda—has been conceptualised by the ILO as “recruitment carried out within the law, in line with international labour standards, and with respect for human rights, without discrimination and protecting workers from abusive situations” (ibid, referencing ILO, 2016: 2). Whereas the term “ethical recruitment” mainly refers to the issue of ‘brain drain’ and the recruitment of skilled workers from developing countries (e.g. healthcare workers) (see Wickramasekara & Baruah, 2017).

44 This is born out of the document analysis conducted by the authors.

45 IOM CREST (2020).

46 The first stage of the Fair Recruitment Initiative ran between 2014 and 2019. The second phase was launched in April 2021 and runs until 2025.

47 These guidelines were developed at a 2016 Tripartite Meeting of Experts and then subsequently published https://www.ilo.org/wcmsp5/groups/public/---ed_protect/---protrav/---migrant/documents/publication/wcms_536263.pdf.

48 The principles included that no recruitment fees should be paid by workers, that workers’ employment terms and conditions be transparent and outlined in an easily understandable manner, and that all workers should be able to access affordable grievance mechanisms. The guidelines also stipulated the responsibilities of governments, public employment services, recruitment agencies, and employers. The ILO stated that these guidelines and principles sought to “inform the current and future work of the ILO and of other organisations, national legislatures, and the social partners on promoting and ensuring fair recruitment” (ILO, 2019a: 11).

49 This online platform aims “to help protect migrant workers from abusive employment practices” through providing them access to migrant workers’ reviews of recruitment agencies. See ITUC (2018).

50 In partnership with the Canadian government, the Swiss Agency for Development and Cooperation, the US Department of State, and the Province of Quebec.

51 This conference also spurred the creation of a Global Policy Network to Promote Ethical Recruitment which IOM launched in December 2020 and which is a Member State-led collaboration which brings together policy-makers, regulators, and practitioners on issues relating to recruitment regulation and the protection of migrant workers (IOM, 2020b).

52 An IOM representative noted: “At the global level, IOM has led a highly consultative process with state and business actors to agree upon an international definition of ethical recruitment, the IRIS standard. The IRIS standard and the adoption of ILO’s definition of recruitment fees and related costs has brought much needed clarity for all stakeholders” (August 2020). Through IRIS, IOM has fostered close cooperation with an array of actors including the recruitment industry, governments, CSOs, trade unions, IOs, and employers.

53 Participants included government representatives, and representatives from regional and international organisations, such as the IOM, ILO, and OSCE.

54 It should be pointed out here that the IOM’s relationship with business and employer organisations is based on seducing business, not about speaking for business which the ILO does on the basis of its tripartite structure. Business has no institutional role in the IOM which is run for and by governments only.

55 Especially in light of the comments previously made by the ITUC General Secretary at the 2011 GFMD: “the IOM has no normative basis for work and no competency to oversee the implementation of states’ obligations under international migration laws” (ICMC, 2011: 25).

56 This will be examined further in the conclusion.

57 For example, the ILO’s account of the co-organised workshop in Guatemala noted that the session aimed “to support countries in the use of different instruments… such as ILO Fair Recruitment Initiative and the IOM International Recruitment Integrity System (IRIS)” (ILO, 2019b). The ILO also noted that the Guatemala workshop “is part of the activities of the *strategic alliance* between the IOM and the ILO” (ibid, emphasis added).

58 An IOM and IOE document (2015) notes that IRIS is being “closely coordinated” with ILO’s Fair Recruitment Initiative.

59 For example, an IOM representative noted “[ILO’s] General Principles and Operational Guidelines on Fair Recruitment are an absolutely fundamental document that I think we all collectively need to use as a reference point. Building on that work, IOM released in June this year a document called the Montreal Recommendations on Recruitment: A Roadmap towards better recruitment regulation… collectively between the two documents, there is a lot that we can draw on in pushing governments in the right direction to tackle these issues” (stated in October 2020). While another stated that “these [IOM] guidelines sit very neatly with the previous ILO’s general principles for fair and ethical recruitment” (IOM Representative, August 2020).

60 The IOM’s Director General stated in April 2021 that “the pandemic requires us to keep our approach to fair and ethical recruitment because still now today there are people who are being hired and they need to abide by the highest levels of fair and ethical recruitment and that is why, from our side, *we will go on working very closely with the ILO*” (stated at the Global Forum for Responsible Recruitment on 12 April 2021, emphasis added).

61 For example, competition may happen at the headquarter level but collaboration may occur in-country where projects are rolled out.

62 See footnote 32.

63 The IOM’s structure does not offer any channels for direct engagement to non-state actors. Unions can be observers at the annual session of the IOM Council if they wish but they have no influence on agenda setting or programming. However, as the ILO’s tripartite structure enshrines the involvement of workers and employers alongside government representatives, ILO-IOM initiatives thus also have to encompass the perspectives of unions and labour NGOs. For example, as noted by the ILO’s Director General “[You] need to engage multiple actors [on recruitment issues]… from the ILO’s point of view, engaging the recruitment industry, engaging employers as well as organised labour is fundamentally important” (stated at the Global Forum for Responsible Recruitment,12 April 2021).

64 Another key line of inquiry relates to in-country project programming which goes beyond the remit of this paper but deserves to be investigated.
